# Artificial Intelligence for Optimizing Cancer Imaging: User Experience Study

**DOI:** 10.2196/52639

**Published:** 2024-10-10

**Authors:** Iman Hesso, Lithin Zacharias, Reem Kayyali, Andreas Charalambous, Maria Lavdaniti, Evangelia Stalika, Tarek Ajami, Wanda Acampa, Jasmina Boban, Shereen Nabhani-Gebara

**Affiliations:** 1 Pharmacy Department Faculty of Health, Science, Social Care and Education Kingston University London Kingston Upon Thames United Kingdom; 2 Department of Nursing Cyprus University of Technology Limassol Cyprus; 3 Department of Nursing International Hellenic University Thessaloniki Greece; 4 Urology Department Hospital Clinic de Barcelona Barcelona Spain; 5 Department of Advanced Biomedical Science University of Naples Federico II Naples Italy; 6 Department of Radiology Faculty of Medicine University of Novi Sad Novi Sad

**Keywords:** artificial intelligence, cancer, cancer imaging, UX design workshops, Delphi method, INCISIVE AI toolbox, user experience

## Abstract

**Background:**

The need for increased clinical efficacy and efficiency has been the main force in developing artificial intelligence (AI) tools in medical imaging. The INCISIVE project is a European Union–funded initiative aiming to revolutionize cancer imaging methods using AI technology. It seeks to address limitations in imaging techniques by developing an AI-based toolbox that improves accuracy, specificity, sensitivity, interpretability, and cost-effectiveness.

**Objective:**

To ensure the successful implementation of the INCISIVE AI service, a study was conducted to understand the needs, challenges, and expectations of health care professionals (HCPs) regarding the proposed toolbox and any potential implementation barriers.

**Methods:**

A mixed methods study consisting of 2 phases was conducted. Phase 1 involved user experience (UX) design workshops with users of the INCISIVE AI toolbox. Phase 2 involved a Delphi study conducted through a series of sequential questionnaires. To recruit, a purposive sampling strategy based on the project’s consortium network was used. In total, 16 HCPs from Serbia, Italy, Greece, Cyprus, Spain, and the United Kingdom participated in the UX design workshops and 12 completed the Delphi study. Descriptive statistics were performed using SPSS (IBM Corp), enabling the calculation of mean rank scores of the Delphi study’s lists. The qualitative data collected via the UX design workshops was analyzed using NVivo (version 12; Lumivero) software.

**Results:**

The workshops facilitated brainstorming and identification of the INCISIVE AI toolbox’s desired features and implementation barriers. Subsequently, the Delphi study was instrumental in ranking these features, showing a strong consensus among HCPs (W=0.741, *P*<.001). Additionally, this study also identified implementation barriers, revealing a strong consensus among HCPs (W=0.705, *P*<.001). Key findings indicated that the INCISIVE AI toolbox could assist in areas such as misdiagnosis, overdiagnosis, delays in diagnosis, detection of minor lesions, decision-making in disagreement, treatment allocation, disease prognosis, prediction, treatment response prediction, and care integration throughout the patient journey. Limited resources, lack of organizational and managerial support, and data entry variability were some of the identified barriers. HCPs also had an explicit interest in AI explainability, desiring feature relevance explanations or a combination of feature relevance and visual explanations within the toolbox.

**Conclusions:**

The results provide a thorough examination of the INCISIVE AI toolbox’s design elements as required by the end users and potential barriers to its implementation, thus guiding the design and implementation of the INCISIVE technology. The outcome offers information about the degree of AI explainability required of the INCISIVE AI toolbox across the three services: (1) initial diagnosis; (2) disease staging, differentiation, and characterization; and (3) treatment and follow-up indicated for the toolbox. By considering the perspective of end users, INCISIVE aims to develop a solution that effectively meets their needs and drives adoption.

## Introduction

### Background

Cancer offers a unique context for medical decisions because of its diverse forms and disease evolution, as well as the requirement to consider each patient’s illness, their ability to receive medical care, accurate treatment responses, early detection, tumor classification or characterization, prediction of local, recurrent, or metastatic tumor progression, precise assessment of treatment strategies and the follow-up monitoring of cancer. These hindrances persist despite advancements in technology [1].

Medical imaging plays a crucial role in the comprehensive treatment of cancer procedures as it provides valuable insights into the morphology, structure, metabolism, and functions of cancers [2,3]. Notably, medical imaging assists health care providers in defining treatment plans, assessing their effectiveness, and guiding follow-up interventions. The increasing amount and availability of collected data (cancer imaging data) and the development of novel technological tools based on artificial intelligence (AI) and machine learning, provide unprecedented opportunities for better cancer detection and classification, image optimization, radiation reduction, and clinical workflow enhancement [2].

The current imaging methods may be improved by identifying findings that are either detectable or not by the human eye and moving from a subjective perceptual skill to a more objective one [2]. To date, related existing research and innovation initiatives, are only limited to small-scale demonstrations, without adequately being validated for reproducibility and generalizability and without exploring large datasets [4]. Therefore, the INCISIVE project [5-10] has been designed to explore the full potential of AI-based solutions or technologies in cancer imaging. The main outcome of this project is to design and develop an improved AI-based technology to address the ongoing challenges of accurate and early detection of cancer, recurrence, and treatment success or failure.

The design and functionalities of the INCISIVE AI toolbox were developed by incorporating the users’ perspectives and experiences. Therefore, the main objective of this study was to gain a comprehensive understanding of the needs of the users, with a specific focus on health care professionals (HCPs) who would use the INCISIVE AI toolbox. Additionally, insights from HCPs were sought to achieve consensus on crucial features of the toolbox, barriers to implementation, and potential users.

### Overview of the INCISIVE Project

The INCISIVE project [5], funded by the European Union’s Horizon 2020 program across 9 European nations, aims to develop and validate an AI-based toolbox to enhance the accuracy, sensitivity, specificity, interpretability, and cost-effectiveness of cancer imaging methods. The project focuses on breast, prostate, lung, and colorectal cancers [5].

## Methods

### Study Design

This was a 2-phase study conducted concurrently. Phase 1 entailed conducting user experience (UX) design workshops, whereas phase 2 entailed leading a Delphi study with HCPs who were the potential users of the INCISIVE AI toolbox.

### Phase 1: UX Design Workshops for INCISIVE AI Toolbox Potential Users, That Is, HCPs

#### Study Design

A qualitative research approach was used to facilitate UX design workshops across the 5 validation countries of the INCISIVE project (Greece, Cyprus, Spain, Italy, and Serbia), in addition to the United Kingdom, which is also a partner of the INCISIVE project. The workshops followed a structured design thinking [11,12] approach, using various methodological tools to guide participants through the problem-solving process. Techniques such as empathizing with users, defining the problem, brainstorming ideas, prototyping, and testing were used. As the project was in the concept stage, the design thinking method was applied up to the ideate stage, focusing on generating innovative solutions for the development of the INCISIVE AI toolbox for cancer care.

#### Participants and Recruitment

A purposive sampling strategy based on the network of the INCISIVE consortium was used to recruit participants. Eligibility criteria included being a medical professional, specifically a general practitioner, radiologist, oncologist, or nuclear medicine physician. Participants were also required to have no prior involvement or affiliation with the INCISIVE project. Through nominations from the INCISIVE partners, potential participants were invited to the workshops via email, receiving a detailed participant information sheet (PIS), a consent form, and a link to access the workshop meetings. The PIS outlined this study’s objectives and workshop agenda, while the consent form ensured volunteer participation. The participants were required to send their consent forms before conducting the workshops.

#### Data Collection Tool

Different use case scenarios (Multimedia Appendix 1) were prepared to facilitate discussion for each workshop with potential users of the AI toolbox. The use case scenarios focused on the patient journey and aimed to elicit information about practice challenges, needs, design features for the AI toolbox, and the level of AI explainability required for the different services suggested to be offered by the toolbox, which were: initial diagnosis, disease staging and characterization, and treatment and follow-up. The use case scenarios were circulated by the research team among the consortium for feedback and refinement. The definite issues (practice challenges, needs, INCISIVE AI toolbox design features, and the level of AI explainability required from the toolbox across potential services) that emerged during various work packages in the INCISIVE project were included in the workshops.

#### Sample Size

The sample size in this study did not depend on statistical power, but on group dynamics among experts [13]. Group discussions in UX design workshops allowed for the exploration of user’s experiences, concerns, and opinions about specific topics and were distinguished by the explicit use of group interaction to generate rich experiential data. Therefore, this study involved a small number of representative end users in each workshop. This approach ensured that there was adequate time for in-depth discussions when addressing requirements. Importantly, this method followed a qualitative approach that relied on the concept of data saturation rather than on sample size.

#### Data Collection

Data collection took place between August and September 2021. Workshops were conducted via Microsoft Teams in a web-based format. The meeting link was sent via email by the research team. In total, 4 workshops were conducted, 1 workshop for each cancer type targeted by INCISIVE (breast, lung, colorectal, and prostate cancer). The research team facilitated and moderated workshops. Each workshop consisted of a panel of 4 participants. Some members from the INCISIVE consortium joined as observers and were able to ask questions and contribute to the discussion in the workshops via the chat functionality. Each workshop lasted an average of 60-90 (SD 20.90) minutes. The participants were provided with a small presentation about various techniques and terminologies to facilitate discussion about AI explainability during the workshops.

#### Data Analysis

The workshops were audio-recorded and transcribed verbatim for analysis. Transcripts were entered into the NVivo (version 12) software for data organization and management. This was followed by collating, synthesizing, and editing emergent ideas to achieve consistent terminology among items expressing similar ideas. The final step involved grouping the generated ideas and items into emerging categories.

### Phase 2: Delphi Study—Identification and Prioritization of INCISIVE Features, Implementation Barriers, and Potential User Groups

#### Study Design

This phase used a mixed methods approach, specifically a modified Delphi approach. The Delphi approach is a systematic method for obtaining, exchanging, and developing informed opinions on a specific issue or set of issues [14]. In this study, a modified ranking-type Delphi approach was used, which aimed at developing group consensus on the relative importance of INCISIVE features, barriers, and potential user groups [13]. It consisted of four rounds. Round 1 involved administering an open-ended questionnaire to the HCPs (Multimedia Appendix 2). Round 2 entailed circulating the anonymized summaries of responses back to the experts for verification. Rounds 3 and 4 involved distilling the most important items chosen by the participants followed by ranking these items.

#### Participants and Recruitment

HCPs involved in cancer care were included in this phase. The recruitment of HCPs was carried out through nominations by INCISIVE partners, following the same inclusion criteria of the UX workshops. The nominated participants received the necessary documentation, including the consent form and the PIS from the research team, and were required to sign the consent form before starting this study.

#### Sample Size

The sample size in the Delphi method does not depend on statistical power but on group dynamics for achieving consensus among experts [13]. Thus, the Delphi literature recommends 10-18 experts for a panel or group of experts within a specific discipline [13,15].

#### Data Collection and Data Collection Tools

##### Overview

**Figure 1:**
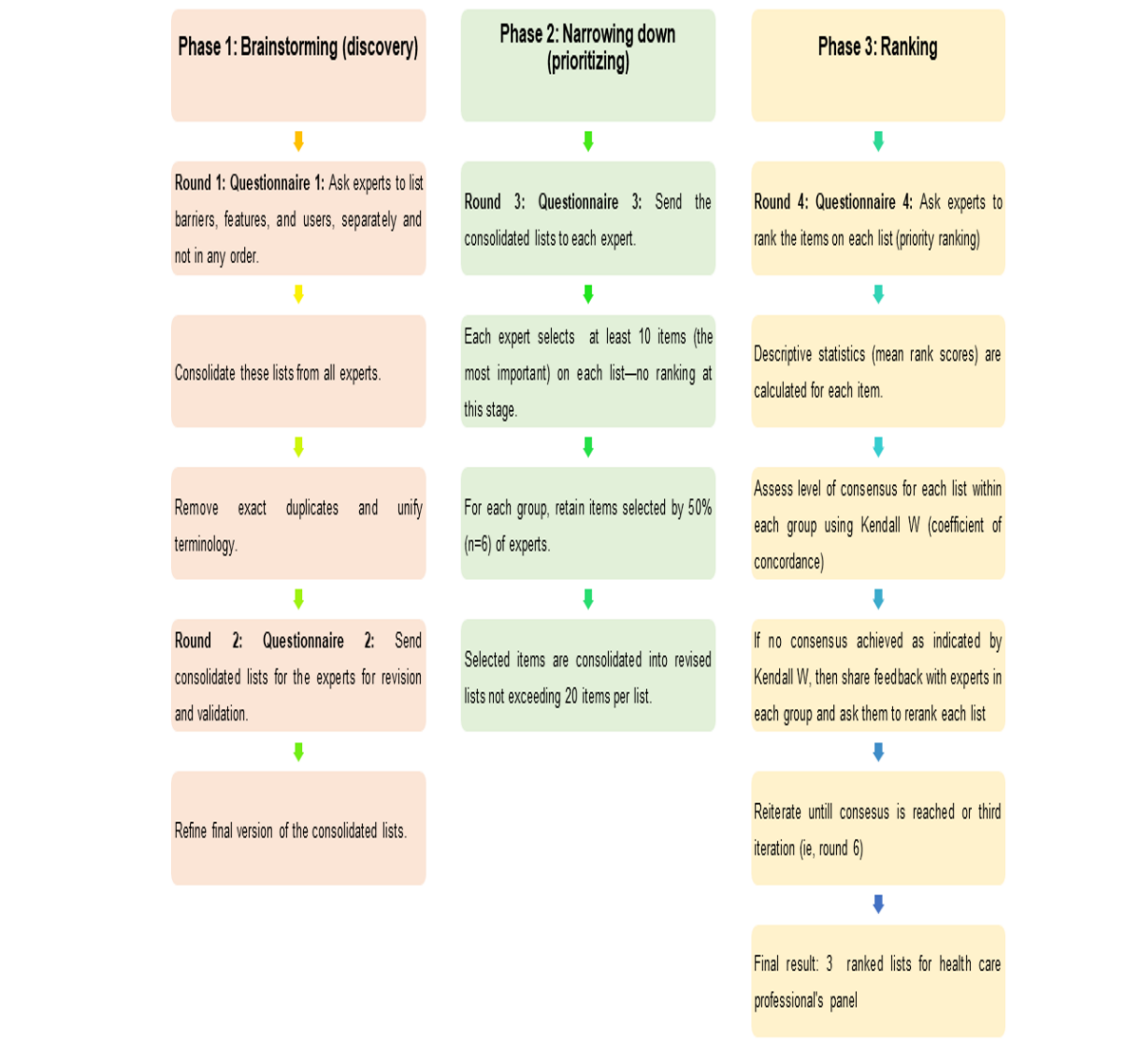
Delphi study administration process (adapted from [8,10]).

Data collection took place between August and September 2021. Delphi is a form of iterative inquiry that builds on ongoing data collection. Its primary research tool is a series of questionnaires built from participants’ stepwise input [15]. Questionnaires were electronically administered via email. The sequence of administration of these questionnaires (ie, data collection) was per the Delphi literature as highlighted in Figure 1 [13,15]. The first questionnaire was sent once the participant agreed to take part and signed the consent form. Questionnaire 1, focused on item generation, required a maximum of 15 minutes to complete, while questionnaires 2 to 4, which involved verification and ranking, took no more than 10 minutes unless participants chose to provide additional explanations for their answers.

##### Questionnaire 1: Generation of Items or Initial Collection of Items

This questionnaire included 3 open-ended questions (Multimedia Appendix 2), about anticipated barriers to the toolbox implementation, essential features required in the INCISIVE AI toolbox, and HCPs who should use the INCISIVE AI toolbox. HCPs were asked to list at least 6 items for each question, followed by a brief explanation of their choices.

##### Questionnaire 2: Validation of Categorized Items

This questionnaire was designed based on the responses obtained from the first round and aimed to strengthen the construct validity according to the concept of “member checking” [15]. This questionnaire included all the consolidated lists obtained from the first questionnaire, with the corresponding categorization. For each list, each item was presented with a brief explanation based on information provided by HCPs in the first round. HCPs were sent questionnaire 2 alongside an exact copy of their responses to the first questionnaire and were asked to (1) verify their responses and confirm that items have been placed in an appropriate category and (2) review the categorizations and suggest refinements or additional items if necessary.

##### Questionnaire 3: Prioritizing Items or Choosing the Most Important Items

Questionnaire 3 presented the refined, consolidated lists produced from questionnaire 2. Each participant was asked to select (not rank) 10 items from each list that they considered the most important.

##### Questionnaire 4: Ranking Items

The questionnaire was designed based on the responses obtained in round 3. The experts were sent the relevant lists with the most important items. Each expert was instructed to rank items in numerical order (importance ranking) by putting the number 1 for the first most important item, 2 for the second most important item, 3 for the third most important item, and so on, with a lower ranking indicating more importance, hence higher ranking. Hence, each expert individually submitted a rank order of the items of each list, one for each of the relevant lists. They were also requested to provide comments justifying their rankings.

#### Data Analysis

#### Questionnaire 1

All data (items and explanations) were entered into the NVivo (version 12) software for data organization and management. The analysis entailed the removal of identical responses, and then collating, synthesizing, and editing the remaining ideas to achieve consistent terminology among items expressing similar ideas. The final step entailed grouping items into emerging categories. As a result, a consolidated preliminary version of the lists with relevant categories was created.

#### Questionnaire 2

Based on responses from questionnaire 1, items were further refined. This resulted in the formation of the final consolidated lists.

#### Questionnaire 3

Items selected by over 50% (n=6) of the experts in the panel were retained. According to the literature, the list size should not exceed 20 items to avoid burdening the participants in the next round [13,15].

#### Questionnaire 4

Descriptive statistics, such as mean rank scores, were calculated to assess the relative importance of items within each list, and the Kendall W coefficient of concordance was used to measure consensus among the experts. The ranking process was repeated until a strong level of agreement (W≥0.7) was achieved or until the third iteration was reached. The research team obtained 3 ranked lists, providing valuable insights and consensus on important aspects of INCISIVE implementation and the AI toolbox.

### Ethical Considerations

Ethical approval for conducting this study was granted by the Research Ethics Committee at Kingston University on August 11, 2021 (reference 2877), for the UX Design Workshops and on August 16, 2021 (reference 2863), for the Delphi study. All other INCISIVE partners did not require any extra layer of ethics for this study. Informed consent forms were provided to participants before the commencement of this study. Participant information was safeguarded through coding, encryption, and secure storage practices. No compensation was provided for study participants. All methods were performed per the Declaration of Helsinki.

## Results

### Phase 1: UX Design Workshops for INCISIVE AI Toolbox Potential Users, That Is, HCPs

In total, 4 workshops were conducted for the INCISIVE AI toolbox; 1 workshop for each cancer type targeted in the project: breast, lung, colorectal, and prostate cancer. A total of 16 HCPs participated in the 4 workshops. Table 1 provides a summary of the participants’ characteristics.

**Table 1:** Characteristics of health care professionals who participated in the INCISIVE AI^a^ toolbox workshops (N=16).

Participants’ characteristics		Number, n
**Gender**		
	Male	8
	Female	8
**Country**		
	United Kingdom	1
	Serbia	1
	Italy	5
	Greece	6
	Spain	1
	Cyprus	1
**Specialty or occupation**		
	General practitioner or doctor	3
	Radiologist	5
	Oncologist	4
	Radiation oncologist, therapeutic radiographer, or radiotherapist	1
	Nuclear medicine physician	2
	Urologist	1

^a^AI: artificial intelligence.

### Features of the INCISIVE AI Toolbox, Irrespective of Cancer Type: Generic Features Required for the INCISIVE AI Toolbox

#### Overview

The section below details the practice challenges, needs, and generic design features required from the INCISIVE AI toolbox across the 3 main potential services.

#### Service 1: Initial Diagnosis

Several challenges were highlighted by the participants at this stage. These included a lack of resources for necessary tests in primary care, especially in rural areas, misdiagnosis, delay in diagnosis, lack of expertise or failure to recognize potential cancer symptoms, and low sensitivity of some imaging modalities. To tackle these issues, the participants envisaged that the INCISIVE AI toolbox can help in several ways including guiding HCPs in primary care in the management and referral of patients mainly in providing a clear protocol on the next steps to be carried out based on the available data at this stage, reduce the chances of misdiagnosis, reduce the chances of overdiagnosis as well avoiding unnecessary anxiety among patients. To promote the efficiency of the pathway, it was discussed that if all HCPs involved in the pathway have access to the INCISIVE AI toolbox, secondary care health professionals can view the tests and images that have already been performed in primary care and take appropriate action to prevent work duplication and loss of time and money. A detailed explanation of this can be found in Multimedia Appendix 3.

#### Service 2: Disease Staging, Differentiation, and Characterization

At this point, several issues were also brought to light, including a lack of resources, particularly imaging equipment, which can cause delays in obtaining the necessary images in a timely manner. Additionally, the proficiency of radiologists in interpreting imaging results and histopathologists in interpreting biopsy results was emphasized as a critical component. Consequently, finding the most accessible, suitable site, or area to do a biopsy, lack of experience among some radiologists and histopathologists, certain imaging modalities such as computed tomography, magnetic resonance imaging, and ultrasound, have low sensitivity, making it difficult for HCPs to distinguish between benign and malignant lesions. The participants anticipated that the INCISIVE AI toolbox would benefit them in several ways, such as enhancing the accuracy of the current imaging tests by identifying small lesions that HCPs might otherwise overlook or lesions that are difficult or confusing for them to identify using the current imaging modalities, assistance with TNM staging and categorization, advice regarding the best places to biopsy, guidance regarding the best imaging tests to run on the patient, support decision-making in cases of disagreement or contradiction of the results generated by the different imaging modalities and tests. For instance, when the results of an imaging test and a biopsy contradict. An extensive overview of this service can be found in Multimedia Appendix 4. The specific features needed for each type of tumor are detailed in Multimedia Appendix 5.

#### Service 3: Treatment and Follow-Up

The challenges in this stage were disease treatment for timing, best treatment options/choices and response, in addition to disease prognosis. Certain participants asserted that treatment options were typically decided on at multidisciplinary team (MDT) board meetings, which could be cumbersome to set up and coordinate per paperwork and board member availability, among other factors. This in return might lead to delay in treatment initiation for patients. Fragmentation of care occurs when HCPs are unable to see or do not have access to the detailed work performed by other HCPs, which is crucial for supporting treatment decisions.

The INCISIVE AI toolkit was envisaged by the participants to be helpful in a variety of ways at this point, such as aiding in the allocation of treatments, serving as a guide for decision support, predicting the prognosis of the disease and the response to treatment, assisting in risk stratification, and supporting MDT board meetings at institutions in both physical and web-based formats. It also enables all MDT board members to access the patient’s holistic profile simultaneously. Thus again, the vision is that the INCISIVE toolbox can support electronic access to patient profiles across the journey thus promoting the integration of care allowing for continuity and efficiency. A detailed description of this service can be found in Multimedia Appendix 6.

### Data Input and Output Requirements of INCISIVE AI Toolbox, Irrespective of Cancer Type

Several input and output requirements were identified for each of the 3 services proposed for the INCISIVE AI toolbox. Interestingly, the participants articulated some suggestions that would make the INCISIVE toolbox more HCP-friendly across the 3 services. The data input and output requirements for the 3 services are summarized in Multimedia Appendix 7.

### Explainable AI: Explainability of the INCISIVE AI Toolbox, Irrespective of Cancer Type

**Figure 2:**
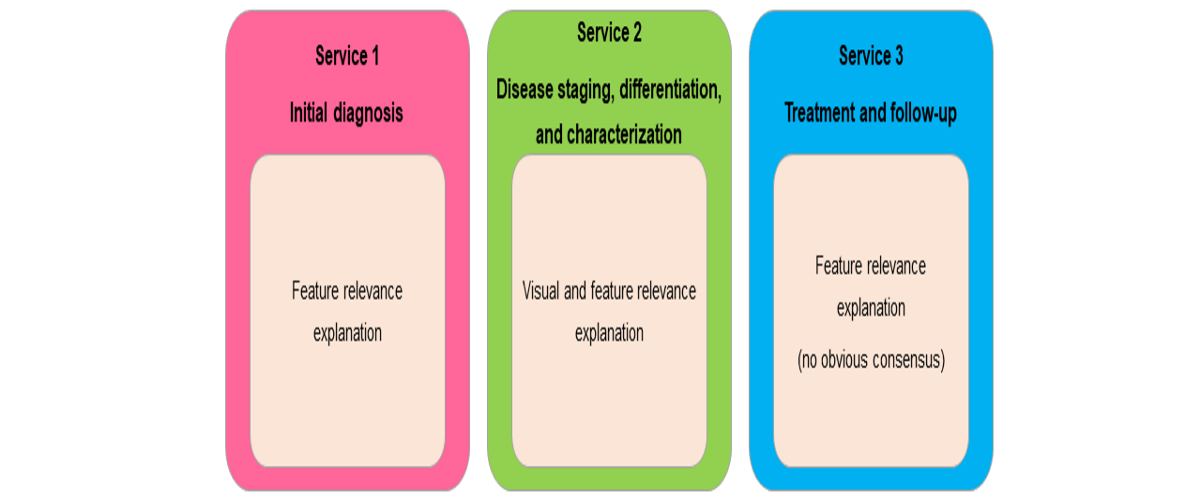
Explainability techniques preference in the INCISIVE AI toolbox across the 3 proposed services. AI: artificial intelligence.

Participants were asked about the explainability techniques they would like to have in the INCISIVE AI toolbox at each stage or service. During the workshops, the participants were prompted with three different explainable AI techniques: (1) feature relevance explanation which attempts to explain a model’s decision by quantifying the influence of each input variable (importance of input features in predicting the output), (2) visual explanation aims at generating visualizations that facilitate the understanding of a model, and (3) explanations by simplification refers to the techniques that approximate an opaque model using a simpler one, which is easier to interpret. Figure 2 explains the options selected by most participants.

### Potential Users or Access to INCISIVE AI Toolbox

#### At Initial Diagnosis

According to the participants, GPs were highlighted as the potential users of the INCISIVE toolbox at this stage and the best HCPs to access and upload information into the system. Some participants highlighted that radiologists would also benefit from having access to the INCISIVE AI toolbox at this stage especially if basic imaging modalities are carried out in primary care, for example, chest x-rays in case of lung cancer.

#### At Disease Staging, Differentiation, and Characterization

Radiologists, pathologists, and nuclear medicine physicians were among the suggested users at this stage. The participants highlighted a very important point which is the need for minimal data input by HCPs to make the INCISIVE AI toolbox as much HCP friendly as possible. About this, some participants suggested assigning the responsibility of data uploading to a nurse or a junior doctor/HCP in order not to increase workload. Nevertheless, the participants envisaged radiologists, pathologists, and nuclear medicine physicians as the most appropriate HCPs for accessing and data processing at this stage. This is because processing images before uploading requires expertise from radiologists and nuclear medicine physicians to identify which images are to be processed and uploaded to the system (ie, the areas of concern) and to identify which parts of the image are to be contoured. The same applies to pathologists for processing histopathological results.

#### At Treatment and Follow-Up

Radiologists, radiation oncologists, oncologists, and surgeons were among the suggested users at this stage. Another interesting finding that emerged out of the 4 workshops was the importance of using INCISIVE at the MDT meetings when deciding treatment options for each patient. According to the participants, all HCPs involved in patient care need to have access to the INCISIVE AI toolbox and to be able to see what other HCPs have performed during the patient’s journey. According to the participants, if the INCISIVE AI toolbox can provide a comprehensive profile for the patient during the MDT meeting including all tests and imaging conducted with the relevant time points, to have all that information in 1 screen, then this would facilitate these meetings to a great extent. Again, these findings are interesting and related to features requested or desired by the participants mainly: the provision of a comprehensive profile for each patient or a complete portfolio and the ability to see the history of all entries carried out by all HCPs involved in the care of the patient.

### Holistic Concerns Emanating From the Workshops

Several concerns were identified throughout the workshops. One main concern was closely intertwined with the minimal data input requirement identified earlier. The concern was related to the amount of time that HCPs will need to dedicate to the INCISIVE AI toolbox. According to participants, currently HCPs are increasingly becoming involved in what they consider nonmedical work (mainly data entry) which is affecting their workload. As such, if the INCISIVE toolbox requires too much data input and attention from HCPs (attention theft) then this would affect HCPs’ willingness to use the proposed toolbox. Another concern was related to the fear that AI technologies such as the INCISIVE AI toolbox can be perceived as a replacement to HCPs in clinical decisions.

### Phase 2: Delphi Study—Identification and Prioritization of Implementation Barriers, INCISIVE Features and User Groups

A total of 12 of the 16 HCPs completed the Delphi study. Participants’ characteristics are summarized in Table 2.

**Table 2:** Characteristics of health care professionals who completed the Delphi study (N=12).

Participants’ characteristics		Participants, n
**Gender**		
	Male	6
	Female	6
**Country**		
	Serbia	2
	Italy	3
	Greece	5
	Cyprus	2
**Specialty or occupation**		
	General practitioner or doctor	1
	Radiologist	4
	Oncologist	3
	Radiation oncologist, therapeutic radiographer, or radiotherapist	1
	Nuclear medicine physician	2
	Surgeon	1

### Features of INCISIVE AI Toolbox

#### Overview

The first and second rounds of questionnaires (questionnaires 1 and 2) involved brainstorming potential features of the INCISIVE AI toolbox and validation. In the first questionnaire, a total of 20 features were generated by the participants and then subsequently validated with no change (via questionnaire 2). In the third round (questionnaire 3) which entailed narrowing down the list, a total of 11 features were retained and prioritized for the INCISIVE AI toolbox. In the fourth round, those 11 features were ranked by importance with a strong consensus among the participating HCPs (W=0.741, *P*<.001; Table 3).

**Table 3:** List of the features in order of importance (priority ranking). A lower mean ranking score indicates a more important feature.

Item importance	Item description	Rank score, mean (SD)
1	Ability to classify the lesion as benign or malignant and the probability of lesion malignancy	2.25 (2.41)
2	Automated lesion spotting and contouring (ie, annotation)	3 (1.9)
3	Automated grading and staging of the disease	3.83 (1.85)
4	Ability to suggest an appropriate course of action during diagnosis and treatment (while keeping the final decision for the clinician)	4.08 (1.37)
5	Ability to link proposed suggestions to established clinical evidence (studies or guidelines)	4.58 (1.72)
6	Ability to predict prognosis	4.75 (1.86)
7	Ability to define response to therapy or treatment	6.92 (1.56)
8	Ability to compare imaging tests and laboratory tests at different time points	7.33 (1.92)
9	Ability to predict the possibility of recurrence	8.75 (1.48)
10	Integration and display of a comprehensive patient profile	9.92 (0.79)
11	Multimodality	10.58 (0.9)

#### Implementation Barriers

HCPs were asked about the barriers that would affect the successful implementation of the AI toolbox proposed by INCISIVE to identify why similar AI solutions usually fail. The first and second rounds of questionnaires (questionnaires 1 and 2) involved brainstorming potential barriers to the successful implementation of the INCISIVE AI toolbox and validation. In the first questionnaire, a total of 23 barriers were identified and then subsequently validated with no change (via questionnaire 2). In the third round, a total of 10 barriers were distilled. In the fourth round, those 10 barriers were ranked by importance with a strong consensus among the participating HCPs (W=0.705, *P*<.001; Table 4).

**Table 4:** List of barriers to the successful implementation of INCISIVE AI^a^ toolbox by importance (with priority ranking). A lower mean ranking score indicates a more important barrier.

Item importance	Item description	Rank score, mean (SD)
1	Lack of resources	1.17 (0.38)
2	Requirement of too much data input from health care professionals	2.75 (2.22)
3	Lack of organizational and management support	3.58 (1.08)
4	Medico-legal issues or concerns: accountability and liability in case of disagreement	4.25 (0.96)
5	Lack of visible advantage of the AI toolbox	5.92 (1.44)
6	Compatibility and integration concerns	6.08 (1.92)
7	Complexity and difficulty of operating the AI toolbox	6.67 (1.37)
8	Concerns related to General Data Protection Regulation (patients’ privacy and confidentiality) and further legal matters in individual countries	6.92 (1.78)
9	Hardware requirements	8.33 (1.77)
10	Data entry bias and variability	9.33 (2.3)

^a^AI: artificial intelligence.

### User Groups for INCISIVE AI Toolbox

The first and second rounds (questionnaires 1 and 2) involved a brainstorming of potential user groups of the INCISIVE AI toolbox and validation. In the first round, a preliminary list of 20 potential user groups was identified. After response validation in the second round, a final consolidated list of 18 potential user groups was identified. In the third round (questionnaire 3) a total of 13 user groups were retained. In the fourth round, those 13 user groups ranked by importance with a strong consensus among the participating HCPs (W=0.767, *P*<.001; Table 5). As expected, higher importance was given to physicians who are common across all tumor types starting from radiologists to nuclear medicine physicians. Whereas lower importance or ranking was provided to tumor-specific HCPs or specialists mainly: pneumologists, gastroenterologists, urologists, and gynecologists.

**Table 5:** The list of INCISIVE AI^a^ toolbox users by priority (with priority ranking). A lower mean ranking score indicates a more important user group.

Item importance	Item description	Rank score, mean (SD)
1	Radiologists	1.5 (0.9)
2	Oncologists	2.5 (1.08)
3	Surgeons (specialized in oncology)	3.42 (1.5)
4	Radiotherapists or radiation oncologists	4.67 (2.77)
5	General medicine practitioners	5.75 (1.76)
6	Multidisciplinary team board	6.17 (2.4)
7	Pathologists	6.58 (1.44)
8	Nuclear medicine physicians	7.83 (1.85)
9	Internists (specializing in oncology)	8.92 (1.5)
10	Pneumologists	10.08 (0.9)
11	Urologists	10.33 (3.33)
12	Gastroenterologists	11.08 (0.51)
13	Gynecologists	12.17 (2.32)

^a^AI: artificial intelligence.

## Discussion

### Principal Findings

The results of this study focused on the specification and prioritization of features guided by the design of the INCISIVE platform. The key findings indicated that the INCISIVE AI toolbox could assist in areas such as misdiagnosis, overdiagnosis, delays in diagnosis, detection of minor lesions, decision-making in disagreement, treatment allocation, disease prognosis, prediction, treatment response prediction, and care integration throughout the patient journey. In addition, the results also provide insight into the implementation barriers that affect the success of solutions such as limited resources, lack of organizational and managerial support, and data entry variability.

The UX design workshops were an answer to many challenges and problems identified. During the stage of initial diagnosis, HCPs highlighted that the toolbox could help in reducing the chances of misdiagnosis and overdiagnosis. Studies highlighted a lack of measures to address diagnostic errors [16,17] and the far-reaching implications of misdiagnosis [18-20] and overdiagnosis [21,22]. The AI toolbox can also guide HCPs in primary care in patient management, thus addressing challenges related to delays in diagnosis, accuracy of imaging modalities, and lack of expertise. During the disease staging, differentiation, and characterization stages, HCPs highlighted that the toolbox could aid in the identification of small lesions that would otherwise be missed by HCPs or lesions that are not very straightforward or easily identified by HCPs, guidance in TNM classification and staging, and the most suitable areas for biopsy, in addition to supporting decisions in cases of disagreement among HCPs or results of the different imaging modalities and tests. HCPs also stressed that the INCISIVE AI toolbox can assist in treatment allocation, disease prognosis prediction, treatment response prediction, and MDT meetings during the third stage of the pathway, which is treatment and follow-up, by addressing issues such as lack of expertise, inaccurate imaging methods, and delays in treatment initiation. An interesting finding emanating from the current work is the vision that AI can support the integration of care across the patient journey, allowing for continuity and efficiency. A feature that proved successful in other chronic conditions in health care [22-24] but has yet to be fully adopted in cancer care in the future.

Several desired features for the INCISIVE AI toolbox were outlined through the Delphi study and the UX design workshops. Interestingly, it can be argued that some of these features apply to the patient’s journey regardless of the journey stage; these include (1) integration and display of a comprehensive patient profile, (2) ability to link proposed suggestions to established clinical evidence (studies or guidelines), (3) ability to check drug interactions, (4) notification of the user of the outcome at each stage, (5) ability to see detailed input from the other HCPs involved in the care of each case, and (6) multimodality. On the other hand, and as highlighted earlier in the results section, some of the features desired by the participants are not feasible within the timeframe of INCISIVE. However, these findings are important and may be considered or viewed within the context of the future sustainability of AI in cancer care.

Some features were commonly identified from the Delphi study and the UX design workshops, and the Delphi study provided a chance to prioritize these features by importance from HCPs’ perspective, which in return would guide the design of the INCISIVE AI toolbox. Mapping of these features against the users’ requirements identified in the INCISIVE project is detailed in Multimedia Appendix 8.

Several barriers were identified to affect the successful implementation of the proposed INCISIVE AI toolbox, thus giving an insight into why similar solutions to the one proposed by INCISIVE usually fail. The participants initially highlighted 23 barriers, which were then distilled down to 10 barriers. Among the most important barriers were lack of resources, lack of organizational and management support, and data entry variability, which are barriers related to the organizational environment. This is not surprising given previous findings in the literature about technology implementation in health care [24]. In previous research by Odeh et al [24] exploring nurses’ perceptions toward a telehealth service, the nurses reported a lack of resources, a lack of organizational support, and a lack of technical support to be among the major issues impacting the service’s implementation. On the other hand, 5 of the 10 barriers were related to the technology itself, mainly hardware requirements, a lack of proven or established advantages of the AI toolbox, compatibility and integration concerns, the complexity and difficulty of operating the AI toolbox, and the requirement of too much data input from HCPs.

The concern expressed by workshop participants about the possible replacement of HCPs if the INCISIVE system or similar technologies proved successful was a noteworthy finding. This apprehension was further echoed in a cross-sectional web-based survey [23] conducted to investigate physicians’ perceptions of Chatbots in health care. Another study [25] has made a positive observation, noting that clinicians demonstrate significant openness when it comes to considering the use of AI-based decision support. This finding emphasizes that AI-based technologies should not be seen as a replacement for HCPs’ expertise in decision-making processes. Instead, it should be regarded as a complementary tool that can assist and augment HCPs’ abilities, ultimately improving the quality and efficiency of health care delivery.

Regarding data input, the HCPs recognized the need for multiple data inputs throughout the patient journey, which can be argued to be essential for creating a holistic personalized profile for each patient. These data inputs include medical history, laboratory results, histopathological results, imaging results, etc. However, during the workshops, one recommendation made by the HCPs was to entrust the duty of data uploading to a nurse or a junior HCP. The remaining 2 barriers were related to medical and legal issues, including medico-legal issues per accountability and liability in case of disagreement and concerns related to General Data Protection Regulation (patients’ privacy and confidentiality) and further legal matters in individual countries. However, this is not new; similar ethical and legal challenges posed by AI in health care have been reported in the literature [26].

Interestingly per the explainability of the proposed AI toolbox, the HCPs expressed interest in having a feature relevance explanation or a hybrid approach that combines feature relevance with visual explanation. This preference aligns with another study [27] that emphasizes the significance of visually directive data-centric explanation methods. In some instances, this preference was driven by specialty and expertise. For instance, during disease staging and characterization (ie, service 2), radiologists were more interested in a visual explanation given their specialty and as a lot of imaging tests take place during this stage of the pathway.

### Strengths and Limitations

This study used both quantitative (Delphi study) and qualitative (UX design workshops) methodologies, which aided in triangulating the data and improved the reliability of the findings. HCPs from a variety of specializations participated in this study from several countries. This diverse perspective is guaranteed to be reflective of a broad spectrum of possible users and situations.

It is also essential to recognize this study’s limitations. This study focused only on the specification and prioritization of features guided by the design of the INCISIVE platform, without taking into consideration what would be defined as success criteria for the overall implementation. Another notable constraint is the lack of a comparison to evaluate if the perspectives about the suggested INCISIVE AI toolkit were better or distinct from those regarding other AI solutions. Due to the limited sample size and geographical representation, the findings may not be universally applicable. The cross-sectional assessment of the user requirements sets the stage for continuous monitoring and evaluation of the user demands across time.

### Conclusions

This paper outlined analysis with regards to the user requirements’ definitions of the INCISIVE system. The current work has identified several features for the INCISIVE AI toolbox that are deemed important to guide in the development of the toolbox. Although some of these features may not be pertinent within the remit and duration of the INCISIVE project, they ensure the sustainability of AI in meeting user needs in the future. These features were prioritized and distilled down according to the universal MoSCoW [28] prioritization technique into 4 categories: “must-have,” “should-have,” “could-have,” and “won’t-have,” or “not have right now” in follow-up research on the INCISIVE project. This step determined the features that would be achievable within the life span of the INCISIVE project and which features are part of the futuristic development of AI in cancer care. Data input and output requirements were also elicited for the INCISIVE AI toolbox. Similarly, these requirements will be prioritized according to the universal MoSCoW prioritization technique to determine what is feasible and can be achieved within the timeframe of the INCISIVE project. Additionally, this paper identified several barriers that would affect the successful implementation of INCISIVE. These barriers will be taken into consideration during the development and implementation phases of the project. Additionally, this paper provided an insight into the level of explainability required from the toolbox and potential users across the 3 services suggested for the toolbox, which are also crucial for guiding the design of the toolbox.

## Appendix

Multimedia Appendix 1 Use case scenarios for AI toolbox users’ workshops. AI: artificial intelligence.

Multimedia Appendix 2 The first Delphi questionnaire for health care professionals.

Multimedia Appendix 3 Practice challenges, needs, and generic features of the INCISIVE AI toolbox at initial diagnosis. AI: artificial intelligence.

Multimedia Appendix 4 Practice challenges, needs, and generic features of the INCISIVE AI toolbox at disease staging, differentiation, and characterization. AI: artificial intelligence.

Multimedia Appendix 5 Specific features required for the INCISIVE AI toolbox at disease staging, differentiation, and characterization. AI: artificial intelligence.

Multimedia Appendix 6 Practice challenges, needs, and generic features of the INCISIVE AI toolbox at treatment and follow-up. AI: artificial intelligence.

Multimedia Appendix 7 Data input and output requirements of INCISIVE AI toolbox. AI: artificial intelligence.

Multimedia Appendix 8 Mapping user requirements related to features of the INCISIVE AI toolbox. AI: artificial intelligence.

## Abbreviations

AI: artificial intelligence
HCP: health care professional
MDT: multidisciplinary team
PIS: participant information sheet
UX: user experience

## Data Availability

The datasets used or analyzed during this study are available from the corresponding author upon reasonable request.
