# Supplementary material for: Artificial Intelligence for Optimizing Cancer Imaging: User Experience Study
Source: JMIR Cancer. 2024 Oct 10;10:e52639. doi: 10.2196/52639 (PMC11502975; doi:10.2196/52639)
Supplement: Multimedia Appendix 1 [file cancer_v10i1e52639_app1.docx]

- **Use case scenario for breast cancer workshop**

INCISIVE is an EU funded project that brings together leading researchers, HCPs and industry partners from across 9 European countries. The main aim of this research project is to develop and validate an AI-based toolbox that enhances the accuracy, specificity, sensitivity, interpretability, and cost-effectiveness of existing cancer imaging methods.

Below we are presenting to you a use case scenario for a patient with breast cancer, and we would like to hear from you how INCISVE would help during the patient’s journey.

| **Initial diagnosis** | **Disease staging, differentiation, and characterisation** | **Treatment and follow-up** |
| --- | --- | --- |
| **Scenario: Part A**  A 38-year-old woman presents to the primary care practitioner (PCP) clinic with a suspicious lump in her right breast.  Upon examination the lump is palpable and unilateral  cervical lymphadenomegaly is discovered​. The PCP consider the symptoms worthy of investigation for possible cancer and decides to request imaging for the neck and the breast. | **Scenario: Part B**  The patient is referred now to your care with the following test results: Ultrasound (US) of the neck describing pathologic lymph nodes unilaterally. On the US of the breast a BI-RADS 5 lesion was detected​. | **Scenario: Part C**  The patient’s tumour is now confirmed as:   - Ductal invasive cancer, G3 - ER negative, PR negative, HER 2 negative. |
| **Questions:**   - What tests and imaging do you usually order/do at this stage? - What are the main challenges that you face at this stage? - How do you see the INCISIVE toolbox working / helping at this stage? - What are the necessary features that you would like to have in the INCISIVE system to help you at this stage? - What type of information do you need in the INCISIVE toolbox? (Data input) - Who needs to be involved/ have access to the INCISIVE toolbox at this stage? - Who is the best person(s) to upload the necessary data/information into the system? - What kind of output would you like to see from the INCISIVE system? - What kind/level of explainability would you like to have in the INCISIVE toolbox at this stage (prompts: feature relevant explanation or visual explanation?) | **Questions:**   - What tests and imaging do you usually order/do at this stage? - What are the main challenges that you face at this stage? - How can the INCISIVE toolbox help at this stage? - What are the necessary features that you would like to have in the INCISIVE system to help you at this stage? - What type of information do you need in the INCISIVE toolbox? (Data input) - Who needs to be involved/have access to the INCISIVE toolbox at this stage? - Who is the best person(s) to upload the necessary data/information into the system? - What kind of output would you like to see from the INCISIVE system? - What kind/level of explainability would you like to have in the INCISIVE toolbox at this stage (prompts: feature relevant explanation or visual explanation?) | **Questions:**   - What tests and imaging do you usually order/do at this stage (during treatment and follow-up)? - How can the INCISIVE toolbox help at this stage? - What are the necessary features that you would like to have in the INCISIVE system to help you at this stage? - What type of information do you need in the INCISIVE toolbox? (Data input) - Who needs to be involved/ have access to the INCISIVE toolbox at this stage? - Who is the best person(s) to upload the data into the system? And who is responsible for follow-up results? - What kind of output would you like to see from the INCISIVE system? - What kind/level of explainability would you like to have in the INCISIVE toolbox (prompts: feature relevant explanation or visual explanation?) |

- **Use case scenario for lung cancer workshop.**

INCISIVE is an EU funded project that brings together leading researchers, HCPs and industry partners from across 9 European countries. The main aim of this research project is to develop and validate an AI-based toolbox that enhances the accuracy, specificity, sensitivity, interpretability and cost-effectiveness of existing cancer imaging methods.

Below we are presenting to you a use case scenario for a patient with lung cancer, and we would like to hear from you how INCISVE would help during the patient’s journey.

| **Initial diagnosis** | **Disease staging, differentiation, and characterisation** | **Treatment and follow-up** |
| --- | --- | --- |
| **Scenario: Part A**  A 72-year-old, ex-smoker patient (45 pack-year smoking history) presents to the primary care practitioner (PCP) clinic with a history of lingering cough on most days for the last 6 weeks. The patient also reports feeling a little out of breath walking up hills and losing a few pounds in weight (~1.5-3 kgs) during the last 6 weeks. The PCP consider the symptoms worthy of investigation for possible cancer and decides to request imaging for the lung. | **Scenario: Part B**  The patient is referred now to your care with the following test results: a chest x-ray indicating the presence of abnormal area in the left lung (opacity in the left lung field). | **Scenario: Part C**  The patient’s tumour is now confirmed as:   - Non-small cell lung cancer (NSCLC): squamous cell carcinoma - PD-L1 positive (≥50%) - Grade 3 - Stage IIIA (the lesion showed only invasion of the mediastinum) - Tumour size: 4cm. |
| **Questions:**   - What tests and imaging do you usually order/do at this stage? - What are the main challenges that you face at this stage? - How do you see the INCISIVE toolbox working / helping at this stage? - What are the necessary features that you would like to have in the INCISIVE system to help you at this stage? - What type of information do you need in the INCISIVE toolbox? (Data input) - Who needs to be involved/ have access to the INCISIVE toolbox at this stage? - Who is the best person(s) to upload the necessary data/information into the system? - What kind of output would you like to see from the INCISIVE system? - What kind/level of explainability would you like to have in the INCISIVE toolbox at this stage (prompts: feature relevant explanation or visual explanation?) | **Questions:**   - What tests and imaging do you usually order/do at this stage? - What are the main challenges that you face at this stage? - How can the INCISIVE toolbox help at this stage? - What are the necessary features that you would like to have in the INCISIVE system to help you at this stage? - What type of information do you need in the INCISIVE toolbox? (Data input) - Who needs to be involved/have access to the INCISIVE toolbox at this stage? - Who is the best person(s) to upload the necessary data/information into the system? - What kind of output would you like to see from the INCISIVE system? - What kind/level of explainability would you like to have in the INCISIVE toolbox at this stage (prompts: feature relevant explanation or visual explanation?) | **Questions:**   - What tests and imaging do you usually order/do at this stage (during treatment and follow-up)? - How can the INCISIVE toolbox help at this stage? - What are the necessary features that you would like to have in the INCISIVE system to help you at this stage at this stage? - What type of information do you need in the INCISIVE toolbox? (Data input) - Who needs to be involved/ have access to the INCISIVE toolbox at this stage? - Who is the best person(s) to upload the data into the system? And who is responsible for follow-up results? - What kind of output would you like to see from the INCISIVE system? - What kind/level of explainability would you like to have in the INCISIVE toolbox (prompts: feature relevant explanation or visual explanation?) |

- **Use case scenario for prostate cancer workshop.**

INCISIVE is an EU funded project that brings together leading researchers, HCPs and industry partners from across 9 European countries. The main aim of this research project is to develop and validate an AI-based toolbox that enhances the accuracy, specificity, sensitivity, interpretability and cost-effectiveness of existing cancer imaging methods.

Below we are presenting to you a use case scenario for a patient with prostate cancer, and we would like to hear from you how INCISVE would help during the patient’s journey.

| **Initial diagnosis** | **Disease staging, differentiation, and characterisation** | **Treatment and follow-up** |
| --- | --- | --- |
| **Scenario: Part A**  A 62-year-old patient presents to the primary care practitioner (PCP) clinic with a history of increased urination frequency coupled with difficulty in urination and feeling of pain and burning during urination. The PCP encounters an enlarged prostate upon digital rectal examination (DRE).  The PCP consider the symptoms worthy of investigation for possible cancer and decides to request specific lab test(s) for the patient. | **Scenario: Part B**  The patient is referred now to your care with the following test results: an elevated level of PSA (13ng/ml) and enlarged prostate as per the DRE. | **Scenario: Part C**  The patient’s tumour is now confirmed as:   - Invasive adenocarcinoma of the prostate​ - Classification cT3b cN0 cM0 (very high-risk group)​ - Metastases: none​ - Gleason Score: 7, grade group 3 |
| **Questions:**   - What tests and imaging (if any) do you usually order/do at this stage? - What are the main challenges that you face at this stage? - How do you see the INCISIVE toolbox working / helping at this stage? - What are the necessary features that you would like to have in the INCISIVE system to help you at this stage? - What type of information do you need in the INCISIVE toolbox? (Data input) - Who needs to be involved/ have access to the INCISIVE toolbox at this stage? - Who is the best person(s) to upload the necessary data/information into the system? - What kind of output would you like to see from the INCISIVE system? - What kind/level of explainability would you like to have in the INCISIVE toolbox at this stage (prompts: feature relevant explanation or visual explanation?) | **Questions:**   - What tests and imaging do you usually order/do at this stage? - What are the main challenges that you face at this stage? - How can the INCISIVE toolbox help at this stage? - What are the necessary features that you would like to have in the INCISIVE system to help you at this stage? - What type of information do you need in the INCISIVE toolbox? (Data input) - Who needs to be involved/have access to the INCISIVE toolbox at this stage? - Who is the best person(s) to upload the necessary data/information into the system? - What kind of output would you like to see from the INCISIVE system? - What kind/level of explainability would you like to have in the INCISIVE toolbox at this stage (prompts: feature relevant explanation or visual explanation?) | **Questions:**   - What tests and imaging do you usually order/do at this stage (during treatment and follow-up)? - How can the INCISIVE toolbox help at this stage? - What are the necessary features that you would like to have in the INCISIVE system to help you at this stage at this stage? - What type of information do you need in the INCISIVE toolbox? (Data input) - Who needs to be involved/ have access to the INCISIVE toolbox at this stage? - Who is the best person(s) to upload the data into the system? And who is responsible for follow-up results? - What kind of output would you like to see from the INCISIVE system? - What kind/level of explainability would you like to have in the INCISIVE toolbox (prompts: feature relevant explanation or visual explanation?) |

- **Use case scenario for colorectal cancer workshop.**

INCISIVE is an EU funded project that brings together leading researchers, HCPs and industry partners from across 9 European countries. The main aim of this research project is to develop and validate an AI-based toolbox that enhances the accuracy, specificity, sensitivity, interpretability, and cost-effectiveness of existing cancer imaging methods.

Below we are presenting to you a use case scenario for a patient with colorectal cancer, and we would like to hear from you how INCISVE would help during the patient’s journey.

| **Initial diagnosis** | **Disease staging, differentiation, and characterisation** | **Treatment and follow-up** |
| --- | --- | --- |
| **Scenario: Part A**  A 66-year-old patient presents to the primary care practitioner (PCP) clinic with a history of diarrhoea and stomach pain on most days for the last 6 weeks. The patient also reported intermittent rectal bleeding, anaemia and weight loss. The PCP consider the symptoms worthy of investigation for possible cancer and decides to request specific lab test(s) for the patient. | **Scenario: Part B**  The patient is referred now to your care with the following test results: a positive faecal immunochemical test (FIT) and suspicious symptoms (as indicated in the report of the PCP). | **Scenario: Part C**  The patient’s tumour is now confirmed as:   - Rectal adenocarcinoma. - Grade 2. - K-RAS positive. |
| **Questions:**   - What tests and imaging do you usually order/do at this stage? - What are the main challenges that you face at this stage? - How do you see the INCISIVE toolbox working / helping at this stage? - What are the necessary features that you would like to have in the INCISIVE system to help you at this stage? - What type of information do you need in the INCISIVE toolbox? (Data input) - Who needs to be involved/ have access to the INCISIVE toolbox at this stage? - Who is the best person(s) to upload the necessary data/information into the system? - What kind of output would you like to see from the INCISIVE system? - What kind/level of explainability would you like to have in the INCISIVE toolbox at this stage (prompts: feature relevant explanation or visual explanation?) | **Questions:**   - What tests and imaging do you usually order/do at this stage? - What are the main challenges that you face at this stage? - How can the INCISIVE toolbox help at this stage? - What are the necessary features that you would like to have in the INCISIVE system to help you at this stage? - What type of information do you need in the INCISIVE toolbox? (Data input) - Who needs to be involved/have access to the INCISIVE toolbox at this stage? - Who is the best person(s) to upload the necessary data/information into the system? - What kind of output would you like to see from the INCISIVE system? - What kind/level of explainability would you like to have in the INCISIVE toolbox at this stage (prompts: feature relevant explanation or visual explanation?) | **Questions:**   - What tests and imaging do you usually order/do at this stage (during treatment and follow-up)? - How can the INCISIVE toolbox help at this stage? - What are the necessary features that you would like to have in the INCISIVE system to help you at this stage at this stage? - What type of information do you need in the INCISIVE toolbox? (Data input) - Who needs to be involved/ have access to the INCISIVE toolbox at this stage? - Who is the best person(s) to upload the data into the system? And who is responsible for follow-up results? - What kind of output would you like to see from the INCISIVE system? - What kind/level of explainability would you like to have in the INCISIVE toolbox (prompts: feature relevant explanation or visual explanation?) |
