# Supplementary material for: Artificial Intelligence for Optimizing Cancer Imaging: User Experience Study
Source: JMIR Cancer. 2024 Oct 10;10:e52639. doi: 10.2196/52639 (PMC11502975; doi:10.2196/52639)
Supplement: Multimedia Appendix 2 [file cancer_v10i1e52639_app2.docx]

**An overview about the INCISIVE project:**

INCISIVE is an EU funded project that aims to develop and validate an Artificial Intelligence (AI)-based toolbox that enhances the accuracy, specificity, sensitivity, interpretability and cost-effectiveness of existing cancer imaging methods. INCISIVE targets the most common types of cancers: lung cancer, breast cancer, prostate cancer and colorectal cancer. Additionally, INCISIVE aims to develop an interoperable pan-European repository of health data including medical images that will enable the secure donation and sharing of data in compliance with ethical, legal and privacy demands, increasing accessibility to datasets and enabling experimentation of AI-based solutions, towards the large-scale adoption of such solutions in cancer diagnosis, prediction and follow-up.

As a HCPs involved in cancer care, we would like to solicit experts’ knowledge and opinion about certain issues related to the INCISIVE project. Therefore, we would really appreciate if you can answer the following questions:

**Open ended questions for HCPs:**

1. List at least six important barriers that you believe would affect the implementation of the INCISIVE AI toolbox. (If possible, please provide a very brief explanation for each barrier)
2. List at least six important features for the INCISIVE AI toolbox. (If possible, please provide a very brief explanation for each feature)
3. List at least six HCPs categories (groups) whom in your experience might play an active role and be the primary users of the INCISIVE AI toolbox within cancer care. For example, these can include general practitioners (GPs), pharmacists, radiologists etc.
