# Supplementary material for: Artificial Intelligence for Optimizing Cancer Imaging: User Experience Study
Source: JMIR Cancer. 2024 Oct 10;10:e52639. doi: 10.2196/52639 (PMC11502975; doi:10.2196/52639)
Supplement: Multimedia Appendix 3 [file cancer_v10i1e52639_app3.docx]

| **Service** | **Practice challenges** | **Needs of INCISIVE AI toolbox** | **INCISIVE AI toolbox design features** |
| --- | --- | --- | --- |
| Initial diagnosis | Lack of resources for the necessary tests in primary care | Guiding HCPs in primary care in the management and referral of patients | 1. Provision of differential diagnosis 2. Use of tick boxes, checklists and prompts to support quick data entry. 3. Provision of a comprehensive profile for each patient / complete portfolio 4. Ability to link the patient’s profile to other family members profiles to extract relevant family history. 5. Ability to suggest next course of action. 6. Ability to see the history of all entries done by all HCPs involved in the care of the patient. 7. Ability of the INCISIVE toolbox to extract patient profile from the institution/hospital database system. 8. Ability to check drug interactions. 9. Ability to check if there is a link between the side effects of current medications taken by the patient and symptoms. 10. Ability to provide visual presentation of lab tests conducted over time. |
|  | Misdiagnosis | Reduce the chances of misdiagnosis. |  |
|  | Delay in diagnosis | Reduce the chances of overdiagnosis |  |
|  | Lack of expertise/failure to recognise potential cancer symptoms | Promoting the efficiency of the pathway |  |
|  | Low sensitivity of some imaging modalities |  |  |
