# Supplementary material for: Artificial Intelligence for Optimizing Cancer Imaging: User Experience Study
Source: JMIR Cancer. 2024 Oct 10;10:e52639. doi: 10.2196/52639 (PMC11502975; doi:10.2196/52639)
Supplement: Multimedia Appendix 4 [file cancer_v10i1e52639_app4.docx]

| **Service** | **Practice challenges** | **Needs of INCISIVE AI toolbox** | **INCISIVE AI toolbox design features** |
| --- | --- | --- | --- |
| **Disease staging, differentiation and characterisation** | Lack of resources (imaging equipment) | Promote the accuracy of the existing imaging tests | - Ability to detect lesion(s). - Ability to identify number of lesions. - Ability to classify lesions as benign or malignant. - Ability to identify tumour(s) borders and volume. - Ability to suggest next course of action: for example, the most appropriate testing and imaging in the form of a checklist to complete the disease staging and differentiation. - Ability to suggest Tumor-Node-Metastasis (TNM) staging (including tumour size and aggressiveness). - Ability to suggest the best site/area for biopsy. - Ability to show/ link up all imaging tests done by the patients (imaging history). - Ability to automatically annotate/contour images and lymph nodes. |
|  | Lack of expertise among radiologists & histopathologists in interpreting the imaging/biopsy results | Guide in TNM classification and staging |  |
|  | Finding the most accessible/suitable site/area to perform biopsy | Guide about the most suitable areas for biopsy. |  |
|  | Low sensitivity of some imaging modalities | Guide about the most appropriate imaging tests to be performed for the patient. |  |
|  |  | Support in decision making in cases of disagreement among HCPs. |  |
|  |  | Support decision making in cases of disagreement or contradiction of the results generated by the different imaging modalities and tests. |  |
