# Supplementary material for: Artificial Intelligence for Optimizing Cancer Imaging: User Experience Study
Source: JMIR Cancer. 2024 Oct 10;10:e52639. doi: 10.2196/52639 (PMC11502975; doi:10.2196/52639)
Supplement: Multimedia Appendix 5 [file cancer_v10i1e52639_app5.docx]

| **Specific features required** | |
| --- | --- |
| **Cancer type** | **Specific features** |
| Breast cancer | - Ability to identify/detect microcalcifications. - Ability to detect extracapsular extension (ECE). |
| Prostate cancer | - Ability to differentiate between clinically significant and non-clinically significant tumours. - Ability to ECE. |
| Lung cancer | - Ability to ECE. |
