# Supplementary material for: Artificial Intelligence for Optimizing Cancer Imaging: User Experience Study
Source: JMIR Cancer. 2024 Oct 10;10:e52639. doi: 10.2196/52639 (PMC11502975; doi:10.2196/52639)
Supplement: Multimedia Appendix 6 [file cancer_v10i1e52639_app6.docx]

| **Service** | **Practice challenges** | **Needs of INCISIVE AI toolbox** | **INCISIVE AI toolbox design features** |
| --- | --- | --- | --- |
| Treatment and follow-up | Challenges with disease treatment in terms of timing, best treatment options/choices and response, in addition to disease prognosis. | Support treatment allocation: decision of the possible treatment choices/ protocols for each patient individually. | - Ability to suggest the possible treatment options for each patient individually. - Ability to suggest the best possible treatment option for each patient individually. - Ability to link up suggested treatment options with clinical evidence from trials supporting each treatment option. - Ability to link up suggested treatment options with relevant guidelines supporting each treatment option. - Ability to assess and predict treatment response/outcome. - Ability to predict disease prognosis. - Ability to have multiple users especially during the MDT board meetings. - Ability to see the history of all entries done by all HCPs involved in the care of the patient. - Ability to predict probability of metastasis in the future. - Ability to predict recurrence. - Ability to compare between sequence of imaging tests (i.e., compare changes in imaging feature during treatment). |
|  | Fragmentation of care as HCPs cannot see or do not have access to the detailed work performed by other HCPs which is crucial to support treatment decision. | Guide in decision (decision support) about the more suitable treatment option for each case individually. |  |
|  |  | Prediction of disease prognosis (prognostic factors). |  |
|  |  | Prediction of treatment response. |  |
|  |  | Support risk stratification. |  |
|  |  | Support individualisation of treatment for each case by itself. |  |
|  |  | Support multidisciplinary team (MDT) board meeting at institutions either virtually or in person, so all MDT board can have access to the holistic profile of the patient at the same time during these meetings. |  |
