# Supplementary material for: Artificial Intelligence for Optimizing Cancer Imaging: User Experience Study
Source: JMIR Cancer. 2024 Oct 10;10:e52639. doi: 10.2196/52639 (PMC11502975; doi:10.2196/52639)
Supplement: Multimedia Appendix 7 [file cancer_v10i1e52639_app7.docx]

| **Service 1: Initial diagnosis** | |
| --- | --- |
| **Data input required** | **Data output required** |
| - Demographics - Patient, family, and medical history - Symptomology - Co-morbidities - Screening history - Lab results - Examination results - Information related to lifestyle - Risk factors (if available) - Extra specific information:   Eg: For breast cancer: history of in-vitro fertilisation | - Differential diagnosis - Checklist of further tests |
| **Service 2: Disease staging, differentiation, and characterisation** | |
| **Data input required** | **Data output required** |
| - Imaging results - Histopathological results - Biomolecular markers - Results of genetic testing (if available) - Imaging parameters   - For prostate cancer: tumour size, ADC (quantitative value of diffusion), capsular infiltration, lymphatic node infiltration, T2 value MRI - For lung cancer: tumour/lesion size, how far is the tumour from bronchus, chest wall invasion | - Number of tumours - Recommendation whether tumour is benign or malignant - Recommendations of further lab and imaging tests required to reach to complete disease staging and differentiation - TNM classification and stage - Presence of ECE in case of prostate, lung, or breast cancers |
| **Service 3: Treatment and follow-up** | |
| **Data input required** | **Data output required** |
| - Patient performance status - Imaging results - Results of molecular testing for prognosis prediction - Treatment results over time - Renal function - Cardiac function | - List of suggested treatment options/protocols for each patient and percentage of prognosis with each treatment option - Recommendation of most appropriate treatment option/protocol for each case - Predictive outcome of treatment employed. - Assessment of treatment response - Probability of metastases in the future for each case - Probability of recurrence in the future for each case |
