# Supplementary material for: Artificial Intelligence for Optimizing Cancer Imaging: User Experience Study
Source: JMIR Cancer. 2024 Oct 10;10:e52639. doi: 10.2196/52639 (PMC11502975; doi:10.2196/52639)
Supplement: Multimedia Appendix 8 [file cancer_v10i1e52639_app8.docx]

| **Initial Diagnosis and Disease Staging and Differentiation** | |
| --- | --- |
| **User requirements** | **Most important and commonly identified features of INCISIVE AI toolbox via the UX design workshop and Delphi study** |
| Reduction in diagnostic delays (reduction of long waiting lists/times for diagnosis) | - Ability to classify the lesion as benign or malignant and probability of lesion malignancy. - Automated lesion spotting and contouring (i.e., annotation). - Automated grading and staging of the disease. - Ability to suggest appropriate course of action during diagnosis (whilst keeping final decision to the clinician). |
| Reduction in chances of misdiagnosis |  |
| Improvement in the accuracy of the current imaging modalities: improve the accuracy of current imaging modalities/tests in terms of reporting and interpretation, rates of false positive and false negative |  |
| Optimisation of resources (addressing lack of HCPs’ expertise) |  |
| Need for improvement in cancer evaluation in terms of characterisation, differentiation and staging |  |
| **Treatment and Follow-Up** | |
| **User requirements** | **Most important and commonly identified features of INCISIVE AI toolbox via the UX design workshop and Delphi study** |
| Reduction in treatment delays (reduction of long waiting lists/times for treatment) | - Ability to suggest appropriate course of action during treatment (whilst keeping final decision to the clinician). - Ability to suggest the best possible treatment option for each patient individually. - Ability to suggest the possible treatment options for each patient individually. - Ability to define response to therapy/treatment. - Ability to compare imaging tests and laboratory tests at different time points. - Ability to predict prognosis. - Ability to predict the possibility of recurrence. |
| Optimisation of resources (addressing lack of expertise and imaging modalities) |  |
| Need for improvement in cancer treatment in terms of timing, choices, and prognosis |  |
| Need for improvement in cancer detection/prediction of cancer recurrence |  |
